# Impact of HIV knowledge and stigma on the uptake of HIV testing – Results from a community-based participatory research survey among migrants from sub-Saharan Africa in Germany

**DOI:** 10.1371/journal.pone.0194244

**Published:** 2018-04-11

**Authors:** Anna Kuehne, Carmen Koschollek, Claudia Santos-Hövener, Adama Thorlie, Johanna Müllerschön, Christina Mputu Tshibadi, Pierre Mayamba, Helene Batemona-Abeke, Stephen Amoah, Virginia Wangare Greiner, Taty Dela Bursi, Viviane Bremer

**Affiliations:** 1 Robert Koch Institute, Department for Infectious Disease Epidemiology, Unit for HIV/AIDS, STI and Blood-borne Infections, Berlin, Germany; 2 Charité University Medicine, Berlin, Germany; 3 Münchner Aids-Hilfe e.V., Munich, Germany; 4 Aidshilfe Essen e.V., Essen, Germany; 5 Pamoja Afrika e.V., Cologne, Germany; 6 Afrikaherz Berlin, Verband für interkulturelle Arbeit, Regionalverband Berlin/Brandenburg e.V., Berlin, Germany; 7 Maisha e.V., Frankfurt am Main, Germany; 8 Hannöversche Aids-Hilfe e.V., Hanover, Germany; University of Miami, UNITED STATES

## Abstract

**Background:**

In 2015, 3,674 new HIV diagnoses were notified in Germany; 16% of those newly diagnosed cases originated from sub-Saharan Africa (sSA). One quarter of the newly diagnosed cases among migrants from sSA (MisSA) are notified as having acquired the HIV infection in Germany. In order to reach MisSA with HIV testing opportunities, we aimed to identify which determinants influence the uptake of HIV testing among MisSA in Germany.

**Methods:**

To identify those determinants, we conducted a quantitative cross-sectional survey among MisSA in Germany. The survey was designed in a participatory process that included MisSA and other stakeholders in HIV-prevention. Peer researchers recruited participants to complete standardized questionnaires on HIV knowledge and testing. We conducted multivariable analyses (MVA) to identify determinants associated with ever having attended voluntary HIV testing; and another MVA to identify determinant associated with having had the last voluntary HIV test in Germany.

**Results:**

Peer researchers recruited 2,782 participants eligible for inclusion in the MVA. Of these participants, 59.9% (1,667/2,782) previously had an HIV test. For each general statement about HIV that participants knew prior to participation in the study, the odds of having been tested increased by 19% (OR 1.19; 95%-CI: 1.11–1.27). Participants reporting that HIV is a topic that is discussed in their community had 92% higher odds of having been tested for HIV (OR 1.92; 95%-CI: 1.60–2.31). Migrants living in Germany for less than a year had the lowest odds of having had their last HIV test in Germany (OR 0.17; 95%-CI: 0.11–0.27). Additionally, MisSA 18 to 25 years (OR 0.55; 95%-CI: 0.42–0.73) and participants with varied sexual partners and inconsistent condom use (OR 0.75; 95%-CI: 0.44–0.97) had significantly lower odds of having had their last HIV test in Germany.

**Discussion:**

Through participatory research, we were able to show that knowledge about HIV and discussing HIV in communities increased the odds of having attended HIV testing among MisSA. However, recent migrants and young sexually active people are among the least reached by testing offers in Germany. Community-based interventions may present opportunities to reach such migrants and improve knowledge and increase discussion about HIV.

## Introduction

At the end of 2015, a total of 36.7 million people were living with HIV globally [1]. Worldwide, 2.1 million people acquired HIV in 2015, 66% in sub-Saharan Africa (sSA), and 4% in Western and Central Europe or North America [1].

In the European Union, 29,747 people were newly diagnosed with HIV and notified in 2015 [2]. Of the newly diagnosed persons, 37% were migrants, 15% from countries with generalized HIV epidemics [2]. People originating from countries with generalized HIV epidemics were more often diagnosed late in the course of the disease (CD4 count < 350/mm^3^) than non-migrants and migrants from other countries [2]. Evidence to suggest acquisition of HIV post-migration and not before has been found in multiple European countries [3].

In Germany 3,674 new HIV diagnoses were notified in 2015 [4]. Of these newly diagnosed cases, 35% originated from countries other than Germany, with 16% originating from sSA [4]. About one quarter to one third of newly diagnosed HIV cases among migrants from sSA (MisSA) were notified as having acquired the HIV infection in Germany [4,5]. This proportion might be an underestimation as research from the United Kingdom indicated that the proportion of cases among migrants having acquired the infection after migration might in fact be three times higher than the notified proportion [6]. The underreporting was identified by comparing physicians’ assumptions of the place of HIV infections with CD4 counts of newly diagnosed cases [6]. Furthermore, among people newly diagnosed with HIV, MisSA were disproportionally affected by AIDS in Germany [4,7]. Research based on German surveillance data combined with a cohort study suggested a “high probability of late presentation and a trend towards later presentation in this group [migrants from high prevalence countries]” and a clear need to identify the barriers to HIV diagnosis amongst migrants in Germany [7].

An HIV test provides a gateway to prevention and care. In Germany, voluntary counselling and testing (VCT) for HIV is available at healthcare facilities, local public health departments (LPHD), and at non-governmental HIV/AIDS counselling organisations. There are no specific HIV testing recommendations for migrants on a national level, but some federal states carry out mandatory HIV screening of asylum seekers upon arrival [8,9].

HIV testing can prevent transmission to sexual partners and children, and early treatment can improve the quality of life and long-term prognosis of people living with HIV (PLHIV) [10]. Uptake of HIV testing is known to be influenced by sex, age, education and income [11,12]. The fear of stigma associated with HIV has also been shown to be associated with uptake of HIV testing, and especially deterred men and young people from HIV testing and treatment in a large qualitative study in South Africa [13]. A systematic review of quantitative studies among PLHIV that analysed the impact of stigma on VCT in low- and middle-income settings concluded that PLHIV who perceived HIV-related stigma as strong had twice as high odds of late presentation for HIV testing than PLHIV who perceived HIV-related stigma as low [14]. A study among MisSA in the United Kingdom found that the driving factors leading people to take an HIV test included a high perceived risk of getting HIV (as opposed to no or low risk) and a previous diagnosis of a sexually transmitted infection (STI) [15].

In order to reduce the number of new HIV infections among MisSA in Germany and to facilitate access to care, attitudes and barriers to VCT among MisSA need to be identified and addressed. Information about MisSAs’ knowledge of and attitudes towards HIV and their VCT uptake in Germany is scarce. This is partly due to the fact that they are not well represented in German HIV behavioural surveys [16] as they are rarely reached with classical study designs that do not sufficiently take into account the views of the groups under study [17–19]. A possibility to include members of the group under study in the research process is the use of community-based participatory health research (CBPHR) [20]. CBPHR involves both members of the group under study and experts from the field in every step of the study process, from study design to data collection and the development of recommendations [20–22]. The involvement of community members improves access for communities and increases understanding of their needs and values [20–23]. CBPHR is a collaborative process that aims to build networks, facilitate mutual learning and the development of relevant recommendations [20,21]. To design adequate HIV prevention messages and to reach MisSA with prevention measures and testing opportunities, information on factors that influence uptake of HIV testing is needed but only partially available [17]. We therefore aimed to identify which determinants influence uptake of HIV testing among MisSA in Germany by using a participatory study design.

## Methods

We conducted a CBPHR cross-sectional multicentre knowledge, attitude, behaviour and practice (KABP) survey on HIV, viral hepatitis and STI among MisSA living in Germany. This publication focusses only on the results regarding HIV-knowledge, -testing uptake and -attitude.

Throughout every step of the CBPHR study process, joint decisions were made by members from MisSA communities in Germany, HIV prevention and testing stakeholders, and researchers. These decisions included aspects relating to study population, study design, questionnaire design, analysis and recommendations. The detailed study protocol, including background information on the methods used in this study, has been published elsewhere [24]. A pilot study was carried out prior to this study in Hamburg, Germany [25].

### Study population

The study population included all MisSA, 18 years of age or above, living in one of the six study sites in Germany, regardless of immigration status. The six study sites were chosen because they had the highest numbers of officially registered MisSA at foreigners’ registration offices at the end of 2013. These include Berlin, Frankfurt, Munich, Cologne, the region of Hannover (including Hildesheim and Brunswick), and the Rhine-Ruhr region (including Duesseldorf, Essen, Oberhausen, Muelheim an der Ruhr and Duisburg). In 2013 officially 43,207 migrants of sSA-nationalities lived in the six study sites, out of a total of 184,440 MisSA registered in Germany [26].

### Study design

We conducted a quantitative cross-sectional survey among MisSA in six study sites between August 2014 and December 2016. The study design was created through a participatory process with an expert group including MisSA living in Germany, and different stakeholders in HIV prevention and researchers and is described in detail elsewhere [24]. We piloted the study design in order to test its feasibility in Hamburg, Germany, and adapted the main study accordingly [25]. Details about the research process that initiated the pilot study, the pilot itself, and the subsequent adaptations made to the study can be found in previous publications [24,25,27].

### Sampling procedure

We used convenience sampling, as a sampling frame that would allow random sampling was not available. Only limited information is available regarding the sSA-population living in Germany [26]. Official statistics only include MisSA with sSA-nationalities, but not those with German citizenship or without regular (legal) status.

MisSA peer researchers with heterogenic ethnical, linguistic, religious and socio-demographic backgrounds recruited a diverse sample of MisSA in their communities. Previous CBPHR studies have shown feasibility and acceptability of participant recruitment through peer researchers, i.e. trained members from the MisSA communities under study [17,28,29]. Peer researchers were selected based on their established contacts with different MisSA communities at the study site and their interest in sexual health topics; full selection criteria are available in the study protocol [24]. In order to recruit a heterogeneous sample of MisSA living in the respective study site, we aimed to adequately represent the biggest subpopulations by countries of birth among the group of peer researchers. In total, 99 peer researchers (10–25 per site) recruited participants over a period of ten to twelve weeks in each study site. During recruitment, we analysed the sociodemographic characteristics of the participants on a weekly basis and provided timely feedback to the local study coordinator and peer researchers about the recruited participants’ sex, age, country of birth, religion, length of stay in Germany, health insurance status, and level of education. We aimed to represent the sSA community in each study site within our sample with regards to known characteristics such as sex and nationality [26]. The peer researchers adapted their future recruitment according to the weekly feedback to assure the sample represented the MisSA community of the study site with respect to sex and nationality and was diverse with regards to other characteristics. This practice also allowed the collection of a heterogeneous sample regarding the other characteristics mentioned above. For example, if the weekly feedback revealed a very small proportion of Muslims in the sample, the peer researchers with appropriate background increased recruitment in mosques.

### Sample size

We aimed to detect differences in proportions of 10% (45% vs. 55%) between men and women with a significance level of 0.05, accepting a beta-error of 0.2. Additionally, migrants from southern Africa, the smallest groups among all MisSA in Germany (about 10%), should make up at least 8.5% of the sample for stratified analysis, accepting a beta-error of 0.2 and alpha-error of 0.05 (both-sided). The total sample size consisting of all six study sites was calculated at 3,009 participants accordingly.

### Data collection

In each study site, an expert group identified a local partner organisation, that was active in HIV prevention among MisSA, and a local study coordinator, MisSA him- or herself. To overcome common language and culture-sampling barriers, the local partner organization selected paid MisSA peer researchers for the recruitment of study participants. Peer researchers received a two-day training on HIV and survey methods, and ethics before participant recruitment started. Peer researchers promoted the study within their community and to other contacts from sSA. Peer researchers recruited participants to fill in a paper-based standardized questionnaire in German, English, or French. The peer researchers obtained consent and either handed out the questionnaire for self-completion or went through the questionnaire with the participant. Participants did not receive any reimbursement for participation in the study. As an incentive, participants received a key chain, a leaflet that outlined local providers of HIV testing and support for PLHIV, and a condom. Questionnaires were sent back in pre-paid envelopes that were addressed to the study coordinators at the Robert Koch Institute (RKI).

The questionnaire was developed collectively by an expert group consisting of MisSA community members, experts working in HIV prevention or HIV clinics, and researchers [24]. Content was based on indicators suggested by the ECDC for behavioural surveillance in migrant populations [30] and the survey instrument of a sexual health survey for African communities from the United Kingdom [11]. The resulting questionnaire was tested prior to the pilot study, and adapted according to the feedback. It was then piloted and adapted again based on feedback from the pilot, pretested again and then finally implemented in this study [24,25,27]. The final questionnaire consisted of 51 questions and 23 knowledge items including: sociodemographic information; HIV, Hepatitis and STI knowledge and testing behaviour; sexual behaviour and risk factors; stigmatization; and preferred means of information. All covered items are available at [24,31].

Items about HIV, Hepatitis and STI knowledge were framed in a way that true information about the respective topic was presented (e. g. “You cannot tell from someone’s appearance whether he or she has HIV or not”) and the participant had the choice to select one of the following answers: “I knew this already”, “I was not sure if that was true or not”, “I didn’t know this,” or “I don’t understand this statement” [24].

### Definitions

Together with the expert group of MisSA and stakeholders in HIV prevention, we defined MisSA as persons who at the time of the study lived in Germany but were born in an sSA country (first generation migrant) or with at least one parent originating from sSA (second generation migrant).

To determine uptake of HIV testing we defined a positive answer as participants who reported having had an HIV test at some point in their life. A negative answer was defined as participants who reported never having had an HIV test or who were not sure if they had ever been tested for HIV.

To determine uptake of HIV testing in Germany we considered participants who stated they had a test for HIV and had their last HIV test in Germany. We did not consider participants who reported to never have had an HIV test, or who were not sure if they were ever tested for HIV, or those who reported that their last test was not in Germany but in their country of origin, in another country, or they did not remember where they were last tested.

For general HIV knowledge, the number of HIV statements that were answered with “I knew this already” was counted, with a possible range of 0–8. The following statements were included: “HIV and AIDS also exist in Germany”, “AIDS is caused by a virus called HIV”, “You cannot tell from someone’s appearance whether he or she has HIV or not”, “There is a test which shows whether someone is HIV positive or not”, “HIV is not transmitted through kissing or shaking hands”, “HIV can be transmitted through sexual intercourse”, “There is no cure for HIV infection”, and “There are medications that can help people with HIV stay healthy”.

Two additional statements on HIV were analysed separately from the count of general HIV knowledge questions, the answers are presented in the descriptive analysis “Africans are NOT deported from Germany just for having HIV” and “In [study site] you can get tested for HIV anonymously and for free, e.g. at the local public health department”. In addition, one question on whether HIV is a topic that is discussed in their communities with the answer options “Yes” and “No” is displayed as it appears in the questionnaire.

To assess HIV related stigma we grouped answers to the question “How would you behave towards a person who is HIV positive?” in two groups. The possible answers to this question included: “I treat them like any other person”, “I avoid physical contact”, “I avoid being seen with this person”, “I blame this person secretly.”, “I behave differently: __”. In the category “I treat persons living with HIV like any other person” all participants that chose the response “I treat them like any other person” were categorized as “Yes” if none of the following statements were chosen in addition “I avoid physical contact”, “I avoid being seen with this person”, “I blame this person secretly.”, “I behave differently: __”. If any of the latter statements were chosen the answer was categorised as “No”.

### Data analysis

All data was entered in Voxco Interviewer Web™ (an online survey and data collection software) and imported to STATA version 14 for analysis.

Participants were eligible to be included in the analysis if they had non-missing and unambiguous answers regarding sex, they or at least one of their parents was born in a sSA-country, and at least 60% of the questionnaire was filled in.

For the descriptive analysis we included only participants that had non-missing and unambiguous answers to the question “Have you ever had an HIV Test?”, i.e. participants that answered “Yes”, “No”, or “I don’t know”. We further excluded participants with missing data on education, length of stay in Germany, and German language skills. We conducted a descriptive analysis on the sociodemographic characteristics, knowledge, attitude, behaviour, and HIV risk factors amongst the study population, and compared those among males and females using a chi-squared test.

We conducted univariable (UVA) and multivariable analyses (MVA) to identify determinants associated with ‘ever having had an HIV test in Germany or abroad’; and a second MVA with the outcome ‘having had the last HIV test in Germany’. For UVA and MVA we excluded participants that reported an HIV test without consent at work, during the visa process, or during the asylum process to ensure that the results were relevant to voluntary uptake of HIV testing. Determinants considered in the UVA and MVA were sociodemographic characteristics (age, sex, education, income, health insurance, religion, length of stay in Germany and German language skills), HIV knowledge (general HIV knowledge, knowledge of the lack of association of HIV diagnosis with deportation, knowledge that LPHD offer testing as defined above) HIV stigma (treating people with HIV like anyone else as defined above and the answer to the statement “HIV is a topic discussed in my community”) and risk factors for HIV (sexual behaviour and STI diagnosis).

### Protection of human subjects

No names, addresses, or other personal identifiers were documented on the questionnaires. Prior to data entry all questionnaires were given a unique identifier. Data protection procedures were cleared by the RKI data protection officer.

All peer researchers received training on the meaning and importance of informed consent. Prior to any recruitment, the study participants were informed of the study objective and voluntary and anonymous nature of the study. The questionnaire was only filled in if informed consent was verbally obtained.

Full ethical clearance was granted for this specific study (full title: “KABP (knowledge, attitudes, behaviour and practices)-study with optional HIV/STI-testing among migrants from sub-Saharan Africa living in Germany (MisSA-study)” by the Ethical Committee of the Charité Berlin in November 2014 (EA4/105/14).

## Results

Peer researchers recruited 3,178 study participants, among them 3,040 were eligible for inclusion in the study. Among the 3,040 participants, 127 were excluded for incomplete information provided in the questionnaires with regards to personal characteristics and HIV testing as described above. Of all included study participants, 53.8% (1,568/2,913) were male. Males and females differed significantly in terms of education, income, health insurance status, religion, length of stay in Germany, uptake of HIV testing, and risky sexual behaviour (Table 1).

**Table 1:** Characteristics of male and female participants of the MisSA study in Germany 2014–2016 (n = 2,913) and differences between male and female participants (chi-squared test).

Characteristics of the study sample		Female (n = 1,345)		Male (n = 1,568)		p-value
		n	%	n	%	
**Sociodemographic characteristics**						
Age						
	Median age in years (range)	31 (18–78)		32 (18–77)		-
Education						
	No school/ primary or secondary school	511	38.0%	480	31.6%	**<0.001**
	Highschool/vocational school	492	36.6%	537	34.2%	
	University/college	342	25.4%	551	35.1%	
Income						
	≤ 1,000 Euro per month	731	54.3%	704	44.9%	**<0.001**
	> 1,000 Euro per month	298	22.1%	508	32.4%	
	Unknown	316	23.5%	356	22.7%	
Health Insurance						
	Regular health insurance	1,132	84.2%	1,235	78.8%	**<0.001**
	No health insurance or medical treatment voucher for asylum seekers from social welfare office	187	13.9%	288	18.4%	
	Unknown	26	1.9%	45	2.9%	
Religion						
	Christian	959	71.3%	980	62.5%	**<0.001**
	Muslim	294	21.9%	431	27.5%	
	No, other or unknown religion	92	6.8%	157	10.0%	
Length of stay in Germany						
	≤ 1 year	148	11.0%	254	16.2%	**<0.001**
	1 - ≤ 5 years	383	28.5%	480	30.6%	
	> 5 years	710	52.8%	745	47.5%	
	Since birth (second generation)	104	7.7%	89	5.7%	
German language skills						
	No or little	342	25.4%	430	27.4%	0.536
	Average or good	588	43.7%	686	43.7%	
	Very good or mothertongue	402	29.9%	444	28.3%	
	Unknown	13	1.0%	8	0.5%	
**Uptake of HIV testing**						
Have you ever had a test for HIV						
	Yes	858	63.8%	915	58.3%	**0.011**
	No	439	32.6%	587	37.4%	
	I don't know	48	3.6%	66	4.2%	
**HIV knowledge and stigma**						
Number of HIV questions answered with "I knew this before"						
	0–4 questions	103	8.0%	145	9.2%	0.135
	5–6 questions	199	14.8%	263	16.8%	
	7–8 questions	1,038	77.2%	1,16	74,0%	
MisSA are not deported from Germany just for having HIV						
	I knew this before	751	55.8%	910	58.0%	0.232
	I did not know/I wasn't sure/unknown	594	44.2%	658	42.0%	
It is possible to get free and anonymous HIV testing at the local public health departments						
	I knew this before	737	54.8%	822	52.4%	0.201
	I did not know/I wasn't sure/unknown	608	45.2%	746	47.6%	
I treat people living with HIV like any other person						
	Yes	968	72.0%	1,121	71.5%	0.667
	No	284	21.1%	325	20.7%	
	Ambiguous answers/unknown	93	6.9%	122	7.8%	
Is HIV/AIDS a topic that is discussed in your community						
	Yes	740	55.0%	919	58.6%	0.124
	No	562	41.8%	597	38.1%	
	Ambiguous answers/unknown	43	3.2%	52	3.3%	
**Risk factors**						
Sex and condom use						
	Had no sex	347	25.8%	345	22.0%	**<0.001**
	Sex with steady partner(s)	633	47.1%	545	34.8%	
	Sex with varied partners/consistent condom use	89	6.6%	188	12.0%	
	Sex with varied partners/inconsistent condom use	208	15.5%	375	23.9%	
	Ambiguous answer/unknown	68	5.1%	115	7.3%	
Have you ever been diagnosed with an STI other than HIV?						
	Yes	148	11.0%	186	11.9%	0.547
	No (my test was negative or I have never been tested)	1,139	84.7%	1,305	83.2%	
	Ambiguous answer/unkown	58	4.3%	77	4.9%	

Overall 60.9% (1,773/2,913) of all participants had ever been tested for HIV. Preferred settings for HIV testing were clinics (67.4%; 1,963/2,913), the LPHD (47.5%; 1,383/2,913), or a HIV testing and counselling centre (33.3%; 970/2,913). A neutral place in the community (6.5%; 190/2,913) was least preferred.

Among all participants, 131 were tested for HIV without their consent during the asylum process, a visa application process, or at work: 3.3% (96/2,913) reported having been tested for HIV without their consent during the asylum process, 0.8% (23/2,913) were tested without their consent during a visa application process, and 0.4% (12/2,913) were tested without their consent for work purposes.

Excluding participants from further analysis who reported testing without their consent for work, visa or asylum purposes (2,913–131 = 2,782) resulted in 59.9% (1,667/2,782) of participants ever having had a voluntary HIV test. Sociodemographic characteristics positively associated with voluntary HIV testing in the multivariable analysis were female sex, higher education attainment, and income of more than 1,000 Euros per month. An age below 26 years and being a second generation migrant were negatively associated with ever being tested for HIV (Table 2).

**Table 2:** Univariable and multivariable analysis for the association between uptake of HIV testing (ever having undertaken an HIV test) and sociodemographic factors, HIV knowledge, stigma and risk factors among participants of the MisSA study in Germany 2014–2016 (excluding testing without consent for visa, asylum and work purposes) (n = 2,782).

Variable		% tested	Univariable Analysis			Multivariable Analysis		
			OR	95%—CI	p-value	aOR	95%—CI	p-value
**Sociodemographic characteristics**								
Sex								
	Men	57.2%	Ref.			Ref.		
	Women	63.0%	1.27	1.09–1.48	**0.002**	1.56	1.29–1.88	**<0.001**
Agegroup								
	18–25 years	35.8%	0.28	0.23–0.35	**<0.001**	0.48	0.38–0.62	**<0.001**
	26–35 years	66.3%	Ref.			Ref		
	36–45 years	72.7%	1.35	1.07–1.70	**0.010**	1.26	0.97–1.64	0.082
	> 46 years	66.4%	1.00	0.78–1.29	0.974	1.01	0.75–1.36	0.944
	Unknown	59.9%	0.76	0.56–1.02	0.070	0.78	0.56–1.10	0.159
Education								
	No school/ primary or secondary school	51.0%	Ref.			Ref.		
	Highschool/vocational school	58.2%	1.34	1.12–1.61	**0.001**	1.37	1.10–1.71	**0.005**
	University/college	71.4%	2.40	1.97–2.92	**<0.001**	1.72	1.34–2.19	**<0.001**
Income								
	≤ 1,000 Euro per month	52.2%	Ref.			Ref.		
	> 1,000 Euro per month	71.1%	2.24	1.86–2.71	**<0.001**	1.47	1.16–1.88	**0.002**
	Unknown	62.8%	1.54	1.27–1.87	**<0.001**	1.42	1.14–1.78	**0.002**
Health Insurance								
	Regular health insurance	61.6%	Ref.			Ref.		
	No health insurance, medical treatment voucher for asylum seekers from social welfare office, unknown	52.3%	0.69	0.56–0.83	**<0.001**	1.11	0.84–1.48	0.445
Religion								
	Christian	65.1%	Ref.			Ref.		
	Muslim	47.4%	0.48	0.41–0.58	**<0.001**	0.66	0.53–0.81	**<0.001**
	No, other or unknown religion	55.8%	0.68	0.52–0.89	**0.005**	1.02	0.74–1.40	0.907
Length of stay in Germany								
	≤ 1 year	53.5%	0.57	0.45–0.72	**<0.001**	1.41	0.98–2.06	0.065
	1 - ≤ 5 years	60.3%	0.75	0.63–0.90	**0.002**	1.23	0.96–1.60	0.105
	> 5 years	66.8%	Ref.			Ref.		
	Since birth (second generation)	20.4%	0.13	0.09–0.18	**<0.001**	0.27	0.17–0.42	**<0.001**
German language skills								
	No, little or unknown	57.0%	0.74	0.61–0.89	**0.001**	1.18	0.92–1.52	0.186
	Average or good	64.2%	Ref.			Ref.		
	Very good or mothertongue	56.2%	0.71	0.59–0.85	**<0.001**	0.88	0.70–1.12	0.316
**HIV knowledge and stigma**								
General knowledge about HIV								
	Number of HIV questions answered with "I knew this before" (8 questions)	-	1.41	1.33–1.49	**<0.001**	1.19	1.11–1.27	**<0.001**
Migrants are not deported from Germany just for having HIV								
	I knew this before	67.6%	2.10	1.80–2.46	**<0.001**	1.37	1.12–1.65	**0.001**
	I did not know/I wasn't sure/unknown	49.8%	Ref.			Ref.		
It is possible to get free and anonymous HIV testing at the local public health departments								
	I knew this before	69.3%	2.34	2.00–2.72	**<0.001**	1.52	1.26–1.84	**<0.001**
	I did not know/I wasn't sure/unknown	49.1%	Ref.			Ref.		
I treat people with HIV like any other person								
	Yes	64.5%	2.49	2.06–3.01	**<0.001**	1.63	1.30–2.03	**<0.001**
	No	42.2%	Ref.			Ref.		
	Ambiguous answers/unknown	64.7%	2.52	1.81–3.50	**<0.001**	2.18	1.48–3.21	**<0.001**
Is HIV/AIDS a topic that is discussed in your community								
	Yes	69.3%	2.63	2.25–3.10	**<0.001**	1.92	1.60–2.31	**<0.001**
	No	46.1%	Ref.			Ref.		
	Ambiguous answers/unknown	64.4%	2.11	1.35–3.31	**0.001**	1.22	0.74–2.01	0.440
**Risk factors**								
Sex and condom use								
	Had no sex	44.7%	0.33	0.27–0.40	**<0.001**	0.45	0.35–0.56	**<0.001**
	Sex with steady partner(s)	71.2%	Ref.			Ref.		
	Sex with varied partners/consistent condom use	61.9%	0.66	0.50–0.87	**0.003**	0.81	0.58–1.12	0.206
	Sex with varied partners/inconsistent condom use	56.5%	0.53	0.42–0.65	**<0.001**	0.72	0.56–0.92	**0.010**
	Ambiguous answer/unknown	52.0%	0.44	0.32–0.60	**<0.001**	0.55	0.38–0.79	**0.001**
Have you ever been diagnosed with an STI other than HIV?								
	Yes	81.1%	3.31	2.46–4.44	**<0.001**	2.14	1.54–2.97	**<0.001**
	No	56.6%	Ref.			Ref.		
	Ambiguous answer/unknown	69.2%	1.73	1.20–1.41	**<0.001**	1.43	0.93–2.20	0.105

For each general statement on HIV that participants knew before the study the odds of having ever been tested increased by 19%. Knowing that migrants cannot be deported from Germany just for having HIV increased the odds by 37%, knowing that testing is available anonymously and free of charge at the LPHD increased the odds by 52% (Table 2). Participants stating they would treat people living with HIV/AIDS the same way they treat other people (as opposed to avoiding physical contact, avoiding being seen with them, or blaming them secretly) had 63% increased odds of being tested. Participants that reported HIV as a topic that is discussed in their community (as opposed to not discussed) had 92% higher odds of having been ever tested for HIV (Table 2). Risky sexual behaviour was negatively associated with ever having undertaken an HIV test; participants that reported varied sexual partners and inconsistent condom use were 28% less likely than participants that reported steady sexual partners to have ever had an HIV test. However, participants that reported a previous STI diagnosis had 2.14-times higher odds of ever having been tested for HIV than those not reporting an STI diagnosis (Table 2).

**Fig 1:**
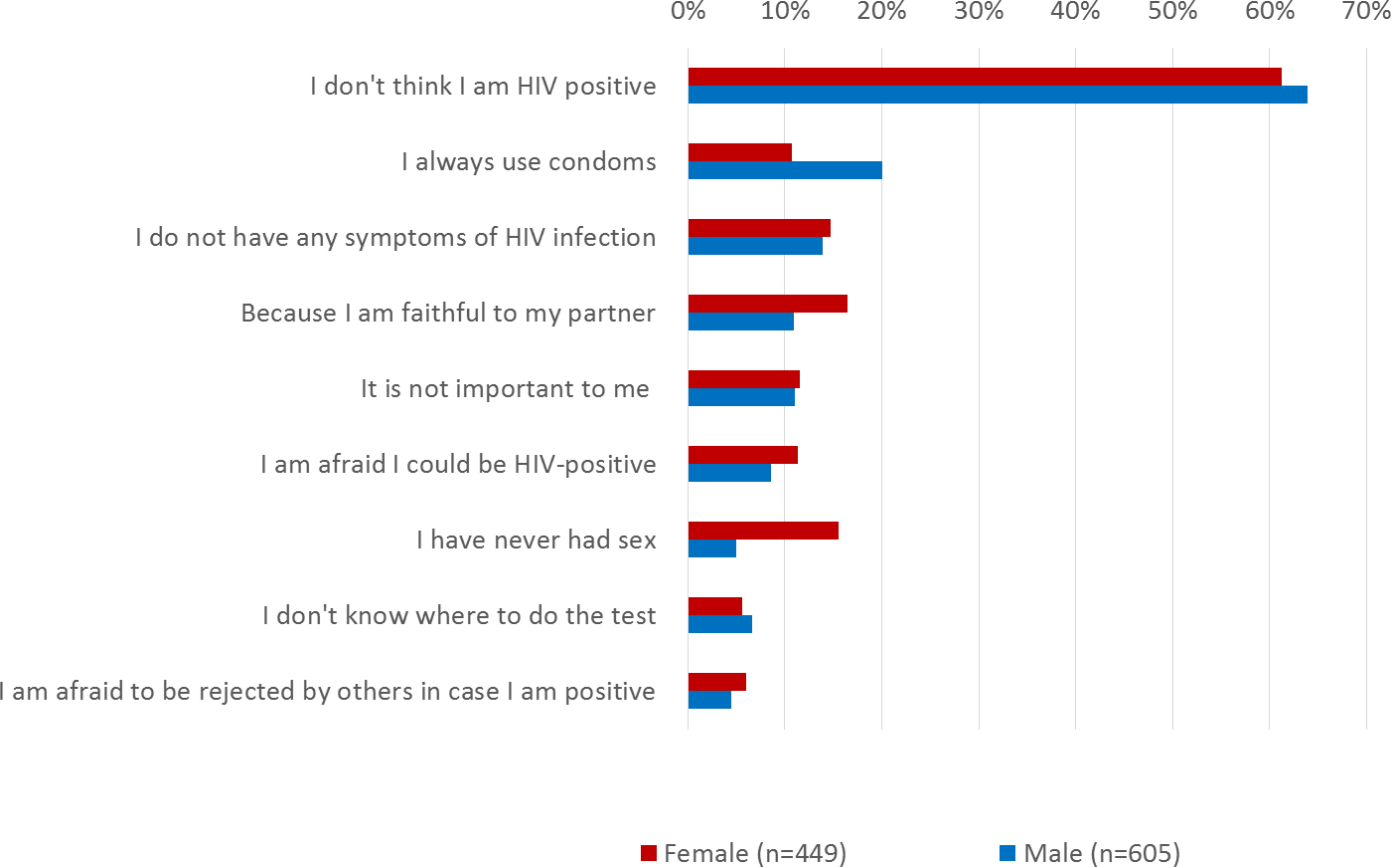
Reasons for not having undertaken an HIV test among the participants of the MisSA study in Germany 2014–2016 (n = 1,054, multiple answers possible).

Among the participants that were never tested for HIV or were not sure if they were tested for HIV, 95% (1,054/1,115) provided reasons for never having undertaken an HIV test. The most frequently mentioned reason was that participants did not think they were HIV positive (female 61%, 275/449; male 64%, 387/605) (Fig 1).

Among the 1,667 participants who had undertaken a voluntary HIV test, 27.5% (458/1,667) had been tested within the past 12 months, 42.0% (700/1,667) in the past one to five years ago, 24.2% (403/1,667) more than five years ago, and 6.3% (106/1,667) did not know when they had last been tested or did not wish to answer.

Among participants tested for HIV, 66.3% (1,106/1,667) reported that their last test had been in Germany, 21.2% (353/1,667) reported the last test had taken place in their country of origin, 7.6% (126/1,667) reported the last test had taken place in another country and 4.9% (82/1,667) did not give an answer.

The multivariable analysis comparing participants who had their last test in Germany (39.8%; 1,106/2,782) to participants who did not have their last test in Germany (including those never tested, unsure if they had been tested and who were last tested elsewhere) indicated a higher odds of having had the last HIV test in Germany for female participants and a lower odds for participants 18–25 years of age (compared to 26–35 years), those with a length of stay in Germany of up to five years (compared to more than five years) and second generation migrants (Table 3).

**Table 3:** Univariable and multivariable analysis for the association between having had the last HIV test in Germany and sociodemographic factors, HIV knowledge, stigma and risk factors among participants of the MisSA study in Germany 2014–2016 (excluding testing without consent for visa, asylum and work purposes) (n = 2,782).

Variable		% tested	Univariable Analysis			Multivariable Analysis		
			OR	95%—CI	p-value	aOR	95%—CI	p-value
**Sociodemographic characteristics**								
Sex								
	Men	34.5%	Ref.			Ref.		
	Women	45.9%	1.61	1.38–1.88	**<0.001**	1.79	1.48–2.15	**<0.001**
Agegroup								
	18–25 years	18.8%	0.33	0.26–0.41	**<0.001**	0.55	0.42–0.73	**<0.001**
	26–35 years	41.5%	Ref.			Ref		
	36–45 years	51.4%	1.49	1.21–1.84	**<0.001**	1.02	0.80–1.31	0.857
	> 46 years	53.5%	1.62	1.28–1.06	**<0.001**	1.02	0.78–1.36	0.846
	Unknown	39.2%	0.91	0.68–1.23	0.536	0.91	0.65–1.28	0.610
Education								
	No school/ primary or secondary school	34.3%	Ref.			Ref.		
	Highschool/vocational school	40.7%	1.31	1.09–1.58	**0.004**	1.04	0.83–1.31	0.708
	University/college	44.4%	1.52	1.26–1.85	**<0.001**	1.09	0.86–1.38	0.490
Income								
	≤ 1,000 Euro per month	31.1%	Ref.			Ref.		
	> 1,000 Euro per month	54.8%	2.70	2.25–3.24	**<0.001**	1.21	0.96–1.51	0.103
	Unknown	40.3%	1.51	1.24–1.83	**<0.001**	1.16	0.93–1.46	0.192
Health Insurance								
	Regular health insurance	43.7%	Ref.			Ref.		
	No health insurance, medical treatment voucher for asylum seekers from social welfare office, unknown	21.5%	0.35	0.28–0.44	**<0.001**	1.16	0.86–1.58	0.327
Religion								
	Christian	43.6%	Ref.			Ref.		
	Muslim	30.4%	0.56	0.47–0.68	**<0.001**	0.85	0.68–1.06	0.143
	No, other or unknown religion	36.8%	0.75	0.57–0.99	**0.044**	0.96	0.70–1.32	0.828
Length of stay in Germany								
	≤ 1 year	10.5%	0.09	0.06–0.13	**<0.001**	0.17	0.11–0.27	**<0.001**
	1 - ≤ 5 years	29.5%	0.33	0.27–0.39	**<0.001**	0.47	0.37–0.60	**<0.001**
	> 5 years	56.2%	Ref.			Ref.		
	Since birth (second generation)	19.9%	0.19	0.13–0.28	**<0.001**	0.36	0.23–0.56	**<0.001**
German language skills								
	No, little or unknown	23.6%	0.37	0.31–0.46	**0.001**	0.89	0.69–1.15	0.371
	Average or good	45.1%	Ref.			Ref.		
	Very good or mothertongue	46.4%	1.05	0.88–1.25	0.585	0.98	0.78–1.23	0.875
**HIV knowledge and stigma**								
General knowledge about HIV								
	Number of HIV questions answered with "I knew this before" (8 questions)	-	1.36	1.28–1.44	**<0.001**	1.12	1.04–1.20	**0.003**
MisSA are not deported from Germany just for having HIV								
	I knew this before	47.6%	2.18	1.86–2.56	**<0.001**	1.30	1.07–1.57	**0.008**
	I did not know/I wasn't sure/unknown	29.4%	Ref.			Ref.		
It is possible to get free and anonymous HIV testing at the local public health departments								
	I knew this before	52.1%	3.18	2.70–3.73	**<0.001**	2.01	1.66–2.43	**<0.001**
	I did not know/I wasn't sure/unknown	25.5%	Ref.			Ref.		
I treat people with HIV like any other person								
	Yes	42.8%	1.95	1.59–2.39	**<0.001**	1.35	1.07–1.72	**0.013**
	No	27.8%	Ref.			Ref.		
	Ambiguous answers/unknown	43.0%	1.96	1.41–2.73	**<0.001**	1.74	1.18–2.56	**0.005**
Is HIV/AIDS a topic that is discussed in your community								
	Yes	43.9%	1.57	1.33–1.84	**<0.001**	1.36	1.13–1.65	**0.001**
	No	33.4%	Ref.			Ref.		
	Ambiguous answers/unknown	44.4%	1.60	1.03–2.47	**0.034**	1.25	0.76–2.5	0.373
**Risk factors**								
Sex and condom use								
	Never had sex	22.4%	0.26	0.21–0.32	**<0.001**	0.42	0.33–0.53	**<0.001**
	Sex with steady partner(s)	52.6%	Ref.			Ref.		
	Sex with varied partners/consistent condom use	40.4%	0.61	0.46–0.80	**<0.001**	0.84	0.61–1.14	0.262
	Sex with varied partners/inconsistent condom use	36.9%	0.53	0.43–0.65	**<0.001**	0.75	0.59–0.95	**0.018**
	Ambiguous answer/unknown	30.5%	0.39	0.28–0.56	**<0.001**	0.65	0.44–0.97	**0.033**
Have you ever been diagnosed with an STI other than HIV?								
	Yes	54.9%	2.03	1.60–2.57	**<0.001**	1.34	1.02–1.77	**0.034**
	No	37.5%	Ref.			Ref.		
	Ambiguous answer/unknown	43.8%	1.30	0.91–1.86	0.147	1.04	0.69–1.57	0.850

For each general statement on HIV that participants knew prior to the study the odds of having had the last test in Germany increased by 12%. Knowing that migrants cannot be deported from Germany just for having HIV increased the odds by 30%, and knowing that testing is available anonymous and free of charge at the LPHD doubled the odds of having had the last HIV test in Germany (Table 3). Participants stating they would treat people living with HIV/AIDS the same way they treat other people (as opposed to avoiding physical contact, avoiding being seen with them, blaming them secretly) had 35% higher odds of having had their last test in Germany. Participants who reported that HIV is a topic that is discussed in their community (as opposed to not discussed) had 36% higher odds of having been tested for HIV the last time in Germany (Table 3). Risky sexual behaviour was negatively associated with a participant’s last HIV test having taken place in Germany; participants that reported varied sexual partners and inconsistent condom use were 35% less likely to have had their last HIV test in Germany than participants that reported steady sexual partners. Participants that reported a previous STI diagnosis had 34% higher odds of having had their last test for HIV in Germany than participants not reporting an STI diagnosis (Table 3).

## Discussion

Sixty percent of participants ever had an HIV test and about two thirds among them had their last test in Germany. Through community-based participatory research, we were able to show that knowledge about HIV and discussing HIV in communities increased the odds of having had an HIV test among MisSA. However, recent migrants and young sexually active people are among the groups with the least uptake of HIV testing services in Germany.

### HIV knowledge and stigma impact on the uptake of HIV testing

As has been shown in previous research, people who have had an HIV test were more often female, older than 25 years, had secondary education or higher, and earned more than 1,000 Euros per month [11,12,15]: Women are tested for HIV as part of antenatal care in many countries and therefore have higher odds of having had an HIV test [12]. Older adults accumulated more possibilities for testing during their lifespan, so their odds of ever having been tested has previously been described to be higher than the odds of younger adults [11,12].

Additionally, we were able to show that participants who declared treating people living with HIV like anyone else–as a proxy for non-stigmatization–had 62% higher odds of having had a test, which corroborates previous findings on the impact of stigma on the uptake of HIV testing [13,14]. Persons that perceived HIV stigma to be high were shown to be more likely to present late to care [14] and were less likely to be successfully linked to care [13]. Furthermore, we found that living in communities that discussed HIV almost doubled the odds of having had an HIV test, i.e. living in communities where HIV is actively addressed seems to have a strong effect on the willingness to get tested.

Participants who had varied sexual partners with inconsistent condom use had 28% lower odds of ever having had an HIV test, a result that might be due to fear of being positive and of being stigmatized. However, there is a possibility that both risky sexual behaviour and having never undertaken an HIV test are due to the same underlying factor, namely lack of knowledge about HIV. We were able to show that each general fact that was known about HIV increased uptake of HIV testing by 19%. Additionally, knowing one could not be deported from Germany just for having HIV increased uptake of HIV testing by 37%; and knowledge of LPHD’s VCT offer increased uptake of HIV testing by 52%. These results are in line with a study in sSA that identified knowledge and education as a driver to increase VCT uptake rather than sexual risk behaviour [32].

### HIV testing in Germany shows least uptake among recent migrants

Among participants that did the last HIV test in Germany, being female was more strongly associated with having had an HIV test, possibly because most female participants were of reproductive age and HIV testing is a standard test offered as part of antenatal care to pregnant woman in Germany [33].

Interestingly, income and education were not associated with having had the last HIV test in Germany, whereas knowing about VCT availability at LPHD doubled the odds of having last had an HIV test in Germany. A possible explanation for these findings could be that VCT is available free of charge for people with no financial means at LPHD in Germany. However, not knowing about the offer of VCT at LPHD decreases the odds of being tested for HIV, indicating that there is a need to increase awareness of LPHD services and eligibility.

Migrants who came to Germany less than a year ago had, among all subgroups, the lowest odds of having had their last HIV test in Germany. This is in accordance to previous findings indicating that recent migrants are less likely to reach (or be reached by) health care as they do not (yet) know where to find preventive and curative care, how to access it, or both [34–36]. Second generation migrants, i.e. migrants who were born in Germany, showed the second lowest odds for having had their last HIV test in Germany. There is possibly less awareness of the risk of HIV infection among second generation migrants than among first generation migrants, as the HIV incidence is higher in sSA countries and more extensive HIV campaigns have been carried out in their countries of origin.

The results indicate that people who had an HIV test in Germany were more likely to be female, older than 25 years, living in Germany for more than five years (but not born in Germany), knew more about HIV, knew that migrants cannot be deported for having HIV, knew where to go for VCT and had sex only with steady partners. While those least reached by HIV testing services in Germany were more likely to be male MisSA, MisSA with a history of risky sexual behaviour, young people, and recent migrants.

Also, an STI diagnosis in the past did increase the odds of having had the last HIV test in Germany, but not to the extent it increased the odds for ever having had an HIV test anywhere. This could indicate that an STI diagnosis in Germany less often prompts the offer of an HIV test than elsewhere, and may present a missed chance for HIV testing.

### Gaps in knowledge and action

The low uptake of HIV testing among young migrants and people with risky sexual behaviour requires innovative interventions that include outreach efforts targeting African communities. Our study shows that discussing HIV increases the uptake of HIV testing; thus, prevention efforts focusing on conversation and de-stigmatization regarding HIV are crucial. Our participatory research project indicates that community-owned intervention can have multiple advantages: a) it increases acceptance of studies or interventions, b) it can reach people in their daily life, in communities that would not be reached by passive offers expecting people to actively go to a certain institution for information, testing, and treatment, and c) it can directly increase the uptake of HIV testing. Previous research has already shown that the active involvement of MisSA community members when planning interventions is highly effective, as their participation increases adoption of preventive measures [37–41].

In addition, the introduction of routine opt-in VCT at clinics, e.g. general practitioner practices, that were mentioned as the preferred option for testing by participants, might increase the uptake of VCT in general–not only among migrants.

Further research will be needed to identify reasons for the very low uptake of HIV testing among second generation migrants in Germany and to identify strategies to improve their uptake of VCT. Involvement of MisSA communities in all steps of the research process will be key to determine research questions that matter to the communities as well as to researchers. The feeling of “ownership” by community members and their collaborative work with peer researchers were shown to be effective in addressing relevant questions with communities and in sparking discussions about HIV with and within the communities [11,15,17,38].

### Limitations

Convenience sampling may have had an impact on representativeness, as may have the weekly feedback and adaptation process during sampling. However, we have no indication that the sample systematically differed from the population with regards to known characteristics of the study population, such as sex and country of origin. However, we do not know how well our sample reflects the local communities and the general MisSA community with regard to unknown characteristics.

We did not apply a standardized instrument to collect information with regards to stigma, as the collective development of a questionnaire with MisSA community members and stakeholders in HIV prevention was given priority over the use of standardized tools. The questions that assessed stigma covered dimensions of fear of infection and social judgement but it did not assess other equally important aspects of HIV-related stigma [42,43].

Recall bias and social desirability bias may further have biased obtained results. We did not use medical records to verify any self-reported information. In addition, the use of true statements (instead of questions) that allowed people to say “I knew this before” may have led to overestimation of knowledge.

As all information was obtained at the same time in this cross-sectional survey, no inference on causality can be made in the MVA with regards to the association between knowledge and behaviour with HIV testing.

We may have underestimated the proportion of those tested in Germany as we only obtained information if the last HIV test was taken in Germany. There is a possibility that participants had not had their last test in Germany but had an HIV test in Germany before.

## Conclusion

One sixth of the cases newly diagnosed with HIV in Germany in 2015 were MisSA [4]. Among cases newly diagnosed with HIV, MisSA were disproportionally affected by AIDS and diagnosed in a late stage of disease [4,7]. In order to improve the uptake of HIV testing among MisSA, we aimed to identify determinants that influence the uptake of testing. Through community-based participatory research, we were able to show that knowledge about HIV and discussing HIV in communities increased the odds of having had an HIV test among MisSA–in addition to known characteristics that are associated with the uptake of HIV testing such as female gender, education and income. People with better knowledge about HIV and people living in communities where HIV is discussed were twice as likely as others to have had an HIV test. However, men, recent migrants, young and sexually active people are among the least reached by HIV testing services in Germany. In order to contact those migrants that are less likely to be reached in HIV testing facilities [13], we recommend community-based interventions [39–41] to increase knowledge and discussion about HIV in migrant communities and thus increase the uptake of HIV testing.
